# Dog Population Rabies Immunity before a Mass Vaccination Campaign in Lima, Peru: Vulnerabilities for Virus Reestablishment

**DOI:** 10.4269/ajtmh.22-0530

**Published:** 2023-07-10

**Authors:** Olimpia Chuquista-Alcarraz, Néstor Falcón, Marco A. N. Vigilato, Felipe Rocha, Gisely Toledo-Barone, Juliana Amorim-Conselheiro, Sergio E. Recuenco, Ricardo Castillo-Neyra

**Affiliations:** ^1^ Unidad Una Salud, Universidad Peruana Cayetano Heredia, Lima, Peru;; ^2^ Facultad de Medicina Veterinaria y Zootecnia, Universidad Peruana Cayetano Heredia, Lima, Peru;; ^3^ Pan American Center for Foot and Mouth Disease and Veterinary Public Health—PANAFTOSA/VPH-PAHO/WHO, Rio de Janeiro, Brazil;; ^4^ Zoonosis Surveillance Division, PAHO/WHO Collaborating Center, Sao Paulo, Brazil;; ^5^ Centro de Investigaciones Tecnológicas, Biomédicas y Medioambientales, Universidad Nacional Mayor de San Marcos, Lima, Peru;; ^6^ Department of Biostatistics, Epidemiology and Informatics, University of Pennsylvania, Philadelphia, Pennsylvania

## Abstract

Lima, Peru, has not had a case of canine rabies since 1999. However, Lima remains at risk of rabies reintroduction due to the free movement of dogs from nearby rabies-endemic areas. In Latin America, rabies vaccination campaigns must reach 80% of dogs to halt transmission, but estimates of vaccine coverage are often unavailable, unreliable, or inaccurate. Quantifying virus neutralizing antibodies (VNA) allows monitoring of the immunological status of the canine population, evaluation of the degree of humoral protection to the virus, and assessing, partially, the population response to vaccination. We evaluated the dog population’s immunity level against the rabies virus before a mass vaccination campaign in Lima. We collected 141 canine blood samples in the district of Surquillo and quantified rabies virus neutralizing antibody titers using the fluorescent antibody virus neutralization test). We surveyed dogs owners to reconstruct canine vaccination histories. Among dogs previously vaccinated, 73.9% exceeded the seroconversion threshold of > 0.5 IU/mL. Among all dogs, only 58.2% reached the titer limit for seroconversion. Dogs ≤ 1 year old constituted 26.2% of the total canine population and had lower levels of VNA than dogs > 1 year old (χ^2^ = 9.071; *P* = 0.028). Importantly, dogs vaccinated with single-pathogen vaccines had higher levels of VNA than those who received combined-pathogen vaccines (χ^2^ = 7.721; *P* = 0.005). We provide an important and timely glimpse to the immunity status of the dog population in urban areas of Lima, a metropolis near a dog rabies–endemic region.

## INTRODUCTION

Rabies vaccination in dogs is a cost-effective, long-term strategy to prevent, control and ultimately eliminate human rabies transmitted by dogs.^1^ In Latin America, the Pan American Health Organization recommends that annual or semiannual canine vaccination campaigns reach coverage levels of 80%^2^^,^^3^ to eliminate rabies.^4^ In Latin America, these campaigns are conducted by public health authorities (i.e., ministries of health) and provided to the population at no cost. Canine herd immunity against rabies prevents not only persistence and transmission of the rabies virus between dogs but also the transmission of rabies virus from other animal species such as bats to dogs and then to people.^5^^–^^8^ Thus, canine herd immunity as an intervention for protecting animal and human health presents a perfect example of a One Health strategy.^9^

The vaccines currently used to prevent rabies in humans and domestic animals are derived from fixed virus genotype 1 (classical rabies virus).^10^ In Peru, rabies vaccines authorized for dogs are inactivated and the vaccine manufactured in Peru is cultured in baby hamster kidney fibroblasts (BHK cells). In recent years, the Peruvian Ministry of Health has been using three types of vaccines in mass dog vaccination campaigns. Vaccines manufactured by the Peruvian National Institute of Health (NIH) have a potency of 1 IU/mL^2^ and Nobivac Rabies and Rabivac Vet vaccines have potencies of 2 and 2.5 IU/mL, respectively.^11^^–^^13^ In Peruvian private veterinary clinics, single-pathogen (monovalent) and combined-pathogen (polyvalent, Biocan) vaccines with different potencies are available. Combined vaccines consist of various pathogen-specific antigens (e.g., leptospirosis, hepatitis, canine distemper), which also include the rabies vaccine.

The level of rabies antibodies found in vaccinated dogs is influenced by the potency of the vaccine, the route of administration, previous exposure to the antigen (i.e., booster), and health of the animal, among other factors.^5^ The antibody level considered adequate is ≥ 0.5 IU/mL, which is the threshold used for the evaluation of rabies vaccines.^14^ It is assumed that immunity conferred by rabies vaccines will be strong and adequate while antibodies remain above this 0.5 IU/mL threshold.^15^ However, it has been shown that dogs with virus neutralizing antibody (VNA) levels below the 0.5 IU/mL threshold might still be protected against rabies virus infection based on B- and T-cell memory response^16^^,^^17^; providing a booster dose reactivates the immune cascade.^18^^,^^19^ In the case of the rabies vaccines used in massive dog vaccination campaigns in Peru and other low- and middle-income countries, this drop in antibodies occurs relatively quickly, necessitating annual booster shots.^2^^,^^20^^,^^21^

Monitoring population immunity in dogs is a helpful tool to prevent the introduction of the rabies virus to rabies-free areas, or interrupt viral transmission, and work toward the elimination of the rabies virus in endemic areas.^22^ Immunological studies on the protective effects of rabies vaccination in dogs are largely based on the determination of adequate neutralizing antibody levels.^20^^,^^23^^–^^25^ Several criteria must be considered in analyzing these neutralizing antibody levels, including the frequency of titers above the adequate threshold (≥ 0.5 IU/mL) and how these titers are affected by dog-associated variables (e.g., age, sex, size of the animal). In addition, the type of vaccine must be considered because differences in immune response have been associated with single-pathogen versus combined-pathogen vaccines.^26^^,^^27^

Lima, the capital of Peru, is a city of 11 million people, and currently, its health surveillance system has not detected any cases of canine rabies since 1999. Nonetheless, several risk factors threaten the city’s canine rabies-free status. Lima’s accessibility from regions such as Puno and Arequipa, where there is active and continued transmission of canine rabies virus, along with the fact that there is no restriction or monitoring of dog migration between regions increases the risk of reintroduction of the rabies virus.^28^ Despite this threat, surveillance of canine rabies is limited, the canine vaccination coverage is presumed to be low, the city has a large dog population (human-to-dog ratio is assumed to be 5:1), and a high proportion of dogs are free-roaming (either stray or owned). The city also lacks a registry or census for dogs, and there is a general lack of available information about the characteristics of the city’s dog population.^29^^–^^34^ The objectives of this study were to quantify the level of population immunity against the rabies virus in the dog population in Lima, to understand the variability of immunity among this population, and to evaluate the city’s vulnerability to the reestablishment of canine rabies virus transmission.

## MATERIALS AND METHODS

### Study site.

This study was conducted in Surquillo district of Lima, Peru’s capital. Surquillo lies at 105 m above sea level with temperatures ranging from 14°C to 26°C.^35^ Environmental issues in Surquillo, as in other districts in Lima, include open dumping of trash, a growing free-roaming dog population (owned and stray), and associated dog fecal contamination and dog bites.^29^

### Study design.

We conducted a cross-sectional serological study during a mass dog vaccination campaign to measure rabies antibody levels in dogs immediately before the annual mass vaccination campaign. To evaluate antibody levels, each dog was bled only once before receiving its vaccine. We used a standardized questionnaire for dog owners to collect data including demographic characteristics (e.g., age, sex, breed, age of dog), vaccination information (e.g., type of vaccine received, vaccination status, having received single or multiple vaccine doses, vaccination location, and years since last vaccination), and address of the owners. For this study, we classified the dog size as small, medium, or large according to the American Kennel Club standards.^36^ Additionally, we classified the type of rabies vaccine (single-pathogen versus combined-pathogen vaccine) according to vaccination certificates.

The mass vaccination campaign was conducted in August 2019; dog owners were verbally invited to participate in the study, and dogs were bled just once before vaccination. After the dog owners signed an informed consent, veterinarians conducted the survey and obtained only one blood sample from the dogs following the recommendations of the World Organization for Animal Health’s Office International des Epizooties (OIE) Biosecurity Manual.^37^ Samples were stored at –20°C and shipped to the laboratory of the Zoonosis Surveillance Division in São Paulo, Brazil. Rabies antibody titration was conducted using a fluorescent antibody virus neutralization, following the recommendations described in the procedure manual for the diagnosis of rabies from the OIE.^38^^,^^39^ Briefly, the test measures the ability of the serum to neutralize the challenge virus. A test that produces fluorescence is considered positive, and the titer level is calculated with graphic or statistical methods. For the rabies virus, international organizations of public health and animal health consider ≥ 0.5 IU/mL the minimum adequate level; titers below this level would indicate inefficacy of the vaccine and/or susceptibility to infection.^5^^,^^22^^,^^38^^,^^39^

### Statistical analysis.

We compared study variables with dogs’ rabies vaccination history. We used the chi-square test to compare categorical variables with five or more observations per group and used the Fisher exact test to compare categorical variables with fewer than five observations in each subgroup. We graphically compared the effect of dog-associated and vaccine-mediated variables on the distribution of titers by using violin plots. We then compared the individual characteristics of the dogs with categorized immune response using chi-square and Fisher exact tests and with linear regression models for titer results. In the bivariate analysis, we used a simple linear regression to evaluate the variable “years since last vaccination” and to determine how antibodies decline over time. Finally, we built a multiple logistic regression model to evaluate the joint effect of individual- and vaccine-level factors on levels of VNA against rabies. All statistical analysis were performed in R version 4.2.0.^4^

## RESULTS

We obtained samples from 141 apparently healthy dogs that were older than 3 months of age and whose owners consented to participate. Among participant owners, 78.7% reported that their dogs had been vaccinated previously (not necessarily during the past year), but only 58% were able to produce the dog’s vaccination certificate. Dogs in the study ranged in age from 3 months to 15 years old (Table 1) and were 48% female. Nine animals were excluded from the study because blood samples were insufficient to obtain serum.

**Table 1:** General characteristics of the study population in terms of rabies vaccination status

Variables	Previously vaccinated % (*N* = 111)	Not previously vaccinated % (*N* = 30)	*P* value (χ^2^)
Age (years)			
≤ 1	12.6 (14)	76.7 (23)	<0.001
> 1–4	45.0 (50)	23.3 (7)	
> 4–7	24.3 (27)	0	
> 7	18.0 (20)	0	
Sex			
Male	49.5 (55)	60.0 (18)	0.418
Female	50.5 (56)	40.0 (12)	
Breed			
Mixed breed	46.8 (52)	76.7 (23)	0.004
Pure breed	53.2 (59)	23.3 (7)	
Size			
Small	37.8 (42)	6.7 (2)	0.005
Medium	55.9 (62)	83.3 (25)	
Large	6.3 (7)	10.0 (3)	
Neutralizing antibody titer			
Adequate levels (≥ 0.5 IU/mL)	73.9 (82)	3.3 (1)	<0.001
Inadequate levels (< 0.5 IU/mL)	26.1 (29)	96.7 (29)	
Vaccination location			
Veterinary clinic	32.4 (36)	–	–
Vaccination campaign	67.6 (75)	–	–
Type of vaccine received			
Combined pathogen	29.7 (33)	–	–
Single pathogen	70.3 (78)	–	–
Vaccination status of dog			
Vaccinated once	24.3 (27)	–	–
Revaccinated	75.7 (84)	–	–

**Figure 1.**
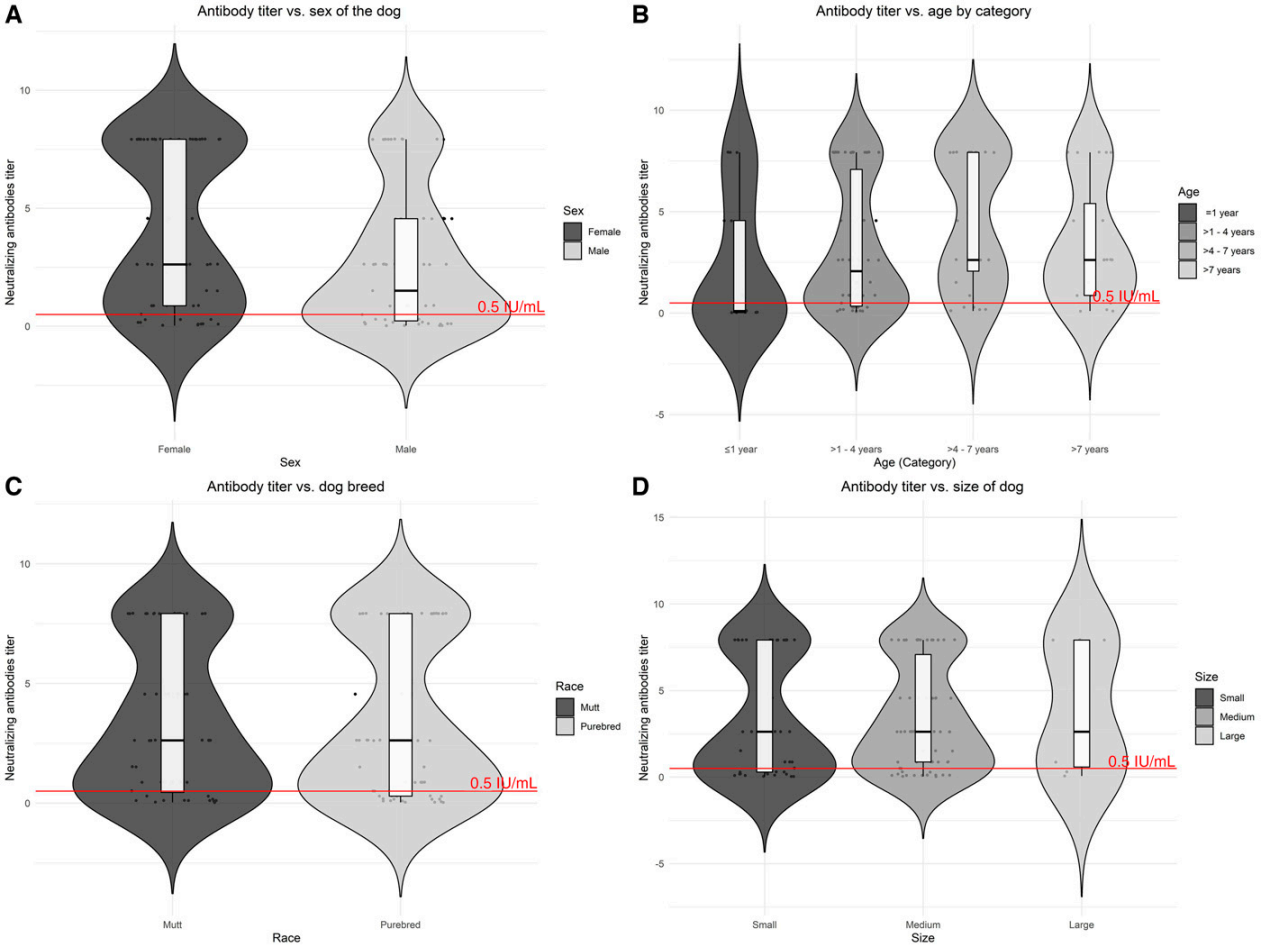
Distribution of antibody titers by dog-level variables. (**A**) Antibody titers by sex of the dogs. (**B**) Antibody titers by dog age group. (**C**) Antibody titers by dog breed. (**D**) Antibody titers by the size of the dog. The red dashed line represents the threshold value (≥ 0.5 IU/mL).

**Figure 2.**
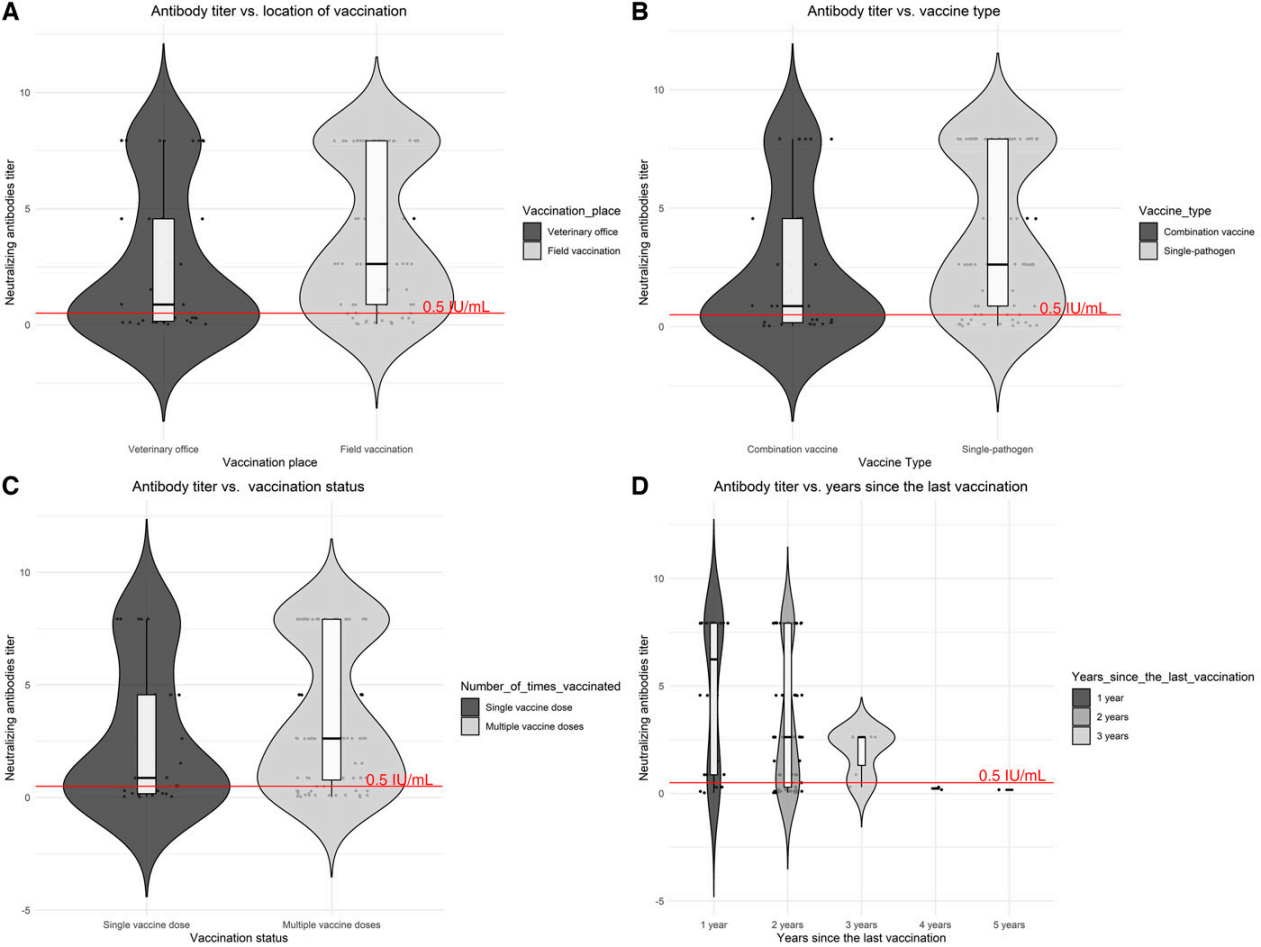
Distribution of rabies antibodies by vaccine-associated variables. (**A**) Antibody titers of dogs that had received a single vaccine dose versus dogs with multiple vaccine doses. (**B**) Antibody titers by vaccine type. (**C**) Antibody titers by vaccination status of the dogs. (**D**) Antibody titers by years since most recent vaccination. The red dashed line represents the threshold value (≥ 0.5 IU/mL).

In our study population, 41.1% of dogs did not have adequate levels of antibody titers. Among dogs that had been previously vaccinated, 73.9% had antibody titers that reached adequate levels (≥ 0.5 IU/mL) (Supplemental Table 1). Titers from dogs that did not reach the threshold value (< 0.5 IU/mL) ranged from 0.03 to 0.49 IU/mL (Supplemental Table 2). The mean titer level was lower in males than in females (2.70 versus 4.15, *P* = 0.016) (Figure 1A). There was not statistical difference in the mean titer level between purebred dogs and mutts (2.69 versus 3.47, *P* = 0.914) (Figure 1C). The mean level titers of dogs vaccinated during vaccination campaigns were higher than dogs vaccinated in veterinary clinics, but the difference was not statistically significant (3.79 versus 2.69, *P =* 0.091) (Figure 2A). Importantly, the mean titer level of dogs vaccinated with single-pathogen vaccines was higher than dogs vaccinated with combined-pathogen vaccine (8.85 versus 2.45, *P =* 0.032) (Figure 2B). The mean titer levels were lower in dogs that had a single vaccine dose than those that had multiple vaccine doses; however, this difference was not statistically significant (2.70 versus 3.79, *P* = 0.238) (Figure 2C), and titer levels were higher in dogs that had been vaccinated in the past 3 years than those who had been vaccinated more than 3 years earlier (3.52 versus 0.21, *P* < 0.001).

**Figure 3.**
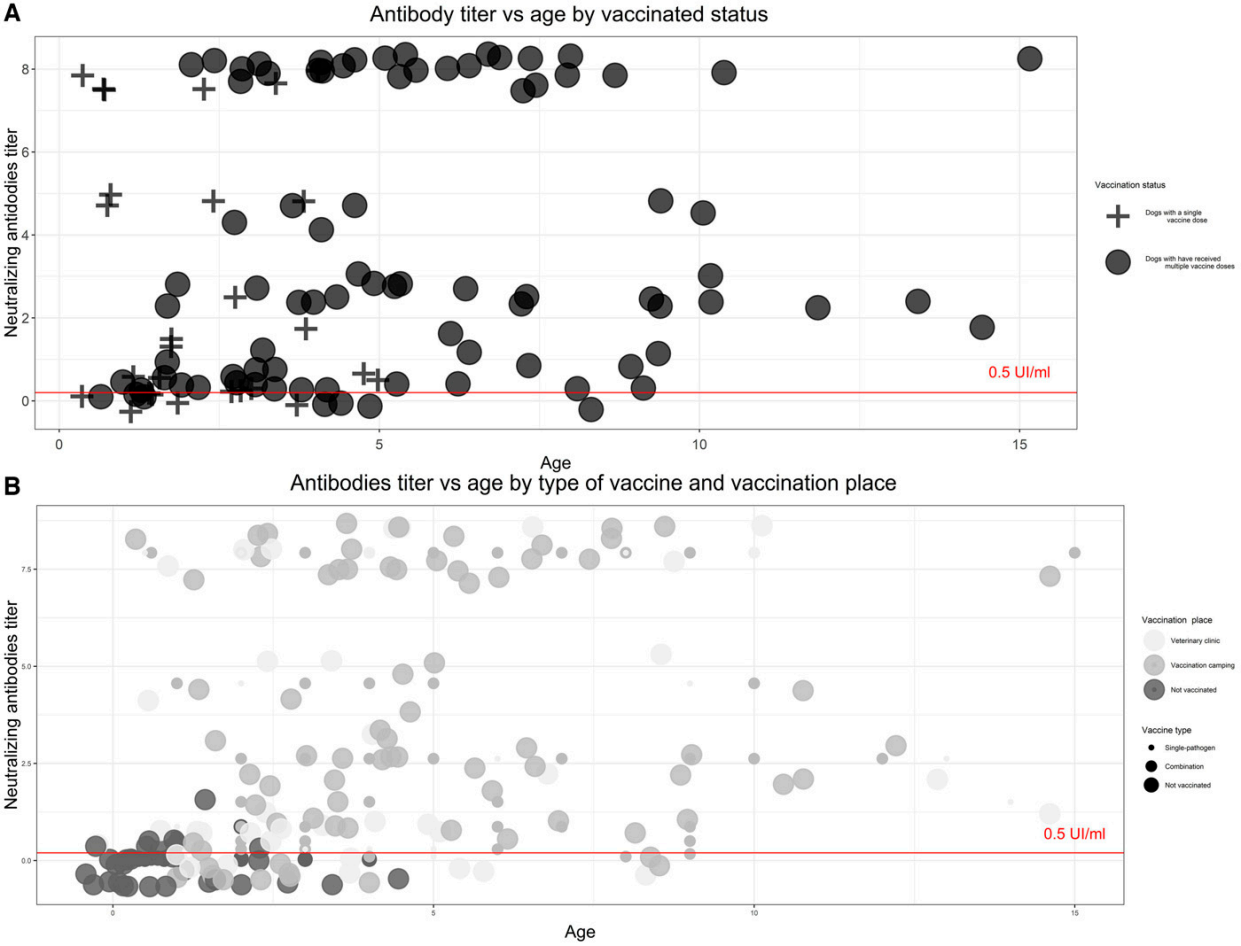
Association between rabies antibody titers and age by vaccine-associated variables. (**A**) Dogs with multiple rabies doses show higher levels of antibodies than dogs with a single vaccine dose and age is associated with receiving multiple vaccine doses. (**B**) Vaccination location and vaccine type do not seem associated with age or antibody titer.

**Figure 4.**
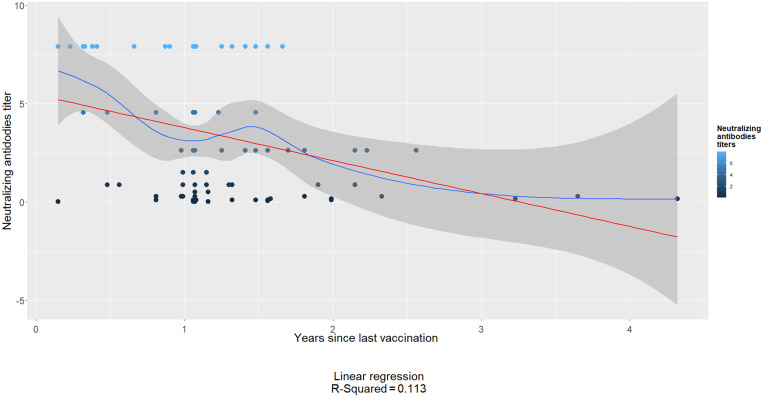
Decline in antibodies as a function of years since last vaccination. The red line is the linear regression between the years since the last vaccination and the level of neutralizing antibodies. The blue line represents the local polynomial regression fitting (loess). The gray area around the blue line is 95% confidence region for the loess regression.

We found that rabies antibody levels were positively associated with the dog’s age (χ^2^ = 9.07; *P =* 0.028), with vaccination location (veterinary office versus field site) (χ^2^ = 7.91; *P =* 0.005), with the type of vaccination received (χ^2^ = 7.72; *P =* 0.005), and with the number of years since the most recent vaccination (χ^2^ = 9.40; *P =* 0.050) (Table 2). Among dogs that had received only one rabies vaccine during their lifetime, only 63% had the minimum acceptable level of antibody titers (Figure 3). There was a negative association between antibody titer levels and years since the most recent vaccination, reflecting the decline in antibodies characteristic of rabies vaccines. Specifically, for each year that passed, antibody titers decreased by an average of 1.68 IU/mL. Nonetheless, only 11% of the variability of antibody titers is explained by years since vaccination (linear regression coefficient of determination = 0.11; Figure 4).

**Table 2:** Dogs characteristics and vaccine variables in the district of Surquillo, Lima, Peru, 2019

Variables	Antibodies result		*P* value (χ^2^)
	Indequate levels	Adequate levels	
	% (*n*)	% (*n*)	
Age (years)			
≤ 1	27.6 (8)	7.3 (6)	0.028
> 1–4	44.8 (13)	45.1 (37)	
> 4–7	17.2 (5)	26.8 (22)	
> 7	10.3 (3)	20.7 (17)	
Breed			
Mixed breed	44.8 (13)	47.6 (39)	0.970
Pure breed	55.2 (16)	52.4 (43)	
Size			
Small	48.3 (14)	34.1 (28)	0.367
Medium	44.8 (13)	59.8 (49)	
Large	6.9 (2)	6.1 (5)	
Sex			
Male	62.1 (18)	45.1 (37)	0.176
Female	37.9 (11)	54.9 (45)	
Vaccination location			
Clinic	55.2 (16)	24.4 (20)	0.005
Campaign	44.8 (13)	75.6 (62)	
Single vs. multiple previous doses			
Single vaccine dose	34.5 (10)	19.8 (17)	0.218
Multiple vaccine doses	65.5 (19)	80.2 (65)	
Type of vaccine			
Combined-pathogen	51.7 (15)	22.0 (18)	0.005
Single-pathogen	48.3 (14)	78.0 (64)	
Years since vaccination			
< 1	17.2 (5)	25.6 (21)	0.052
1 to < 2	69.0 (20)	68.3 (56)	
2 to < 3	3.4 (1)	6.1 (5)	
≥ 3	10.3 (3)	0 (0.0)	

We found that the association of VNA levels with age and being female was still positive and significant after adjusting for other variables (Table 3). Time since last vaccination was negatively associated with VNA levels, with 1.75 less titers for every more year since last vaccination and this association was significant after adjusting for other variables (Table 3).

**Table 3:** Association between levels of virus neutralizing antibodies and individual- and vaccine-level factors

Variables	Multiple linear regression (*N* = 111)		
	Coefficient	95% CI	*P*
Age (years)			
≤ 1	Reference	–	–
> 1–4	0.98	–0.77 to 2.72	0.277
> 4–7	2.66	0.71 to 4.59	0.008
> 7	1.40	–0.66 to 3.47	0.186
Sex			
Male	Reference	–	–
Female	1.39	0.29 to 2.49	0.014
Type of vaccine			
Combined pathogen	Reference	–	–
Single pathogen	0.51	–0.73 to 1.74	0.422
Years since last vaccination	−1.75	–2.63 to −0.86	<0.001

## DISCUSSION

We found that more than 40% of dogs that attended rabies vaccination campaigns in central Lima, Peru, were vulnerable to infection with the rabies virus. Among dogs with any history of past rabies vaccination, 74% had VNA above 0.5 IU/mL, concordant with the expected drop in antibodies levels.^19^^,^^20^^,^^40^ Despite the lack of available data describing canine population dynamics,^41^ our results suggest that a significant proportion of the city’s dog population is likely susceptible or receptive to the introduction of rabies. This threat is probably most serious in peri-urban areas, where coverage levels are usually lower and more dogs are unconfined, resulting in higher contact rates and more rapid population turnover.^42^^,^^43^

Few previous studies have addressed levels of dog rabies immunity in Lima, Peru. In 2004, López reported that 67% of biting dogs evaluated at a rabies health center in Lima had antibody levels > 0.5 IU/mL.^44^ Our results may differ from this 2004 study because the sampling schemes and source populations were unique. Specifically, differences included year of analysis, origin of dogs within the city, and differences in laboratory techniques. The lower VNA levels that we found should not be attributed to vaccine type because the current vaccines are more potent than those used in or before 2004. In 2007, in other areas of Peru, population immunity was estimated to be lower to what we found: 28% of dogs in Piura and 34% of dogs in Puno had levels of antibodies ≥ 0.5 IU/mL 3 months after a mass rabies vaccination campaign.^45^ In other South American countries, the reported post-vaccination seroconversion levels are lower than what we found in Lima: 36% in Montevideo, Uruguay; 50% in Presidente Prudente y Dracena, Brazil; and 40% in San Sebastian de Mariquita, Colombia.^6^^,^^26^^,^^27^^,^^46^

Even though we observed a decay in antibody levels as a function of time, adequate levels of rabies neutralizing antibodies were observed in dogs whose last vaccination was within the past 3 years (Figure 2D). Another study evaluated the number of vaccine boosters and found that dogs that were vaccinated for 3 consecutive years had adequate titers.^26^ It is possible that dogs in our study had not been vaccinated for more than 3 years or were subjects to irregular (not annual) vaccination previous to their last vaccine. Thus, their immunological status not only represents the time since last vaccination but inconsistent vaccination. Interestingly, dogs that received single-pathogen rabies vaccines had higher titers than dogs immunized with combined-pathogen vaccine (*P* = 0.005).

The Ministry of Health in Peru only uses single-pathogen rabies vaccines for their mass vaccination campaigns, whereas private clinics use both single-pathogen and combined-pathogen vaccine, which differ in vaccine strain, adjuvant, and potency.^2^^,^^5^^,^^6^^,^^47^ Our finding of higher titers among dogs vaccinated with single-pathogen vaccines compared with those vaccinated with combined-pathogen vaccine is similar to results from Uruguay and France, where single-pathogen vaccines provided higher rates of serological conversion than combined-pathogen vaccine.^20^^,^^27^ However, Rimal et al. found no differences between the two types of vaccines.^48^ We also found higher titers in dogs vaccinated in public mass vaccination campaigns than dogs vaccinated at private veterinary clinics (*P* = 0.005). This may be related to the type of vaccine used; in Peru, single-pathogen vaccines are used in mass dog vaccination campaigns, whereas combined-pathogen vaccine are commonly used in private veterinary clinics. It is important to note that the vaccines used and produced in Peru by the Peruvian NIH at the time of this study had a potency of 1 IU/mL,^2^ one-third of the concentration of the vaccines used in Brazil^35^ and one-half of the imported vaccines used in Peru.

Dogs younger than 1 year old had on average 44% lower antibody titers than dogs 1 year old or older (Figure 1B); other authors have found a similar association with age.^40^^,^^46^^,^^49^ However, in Colombia, Bonilla et al. found the opposite association—that is, higher antibody titers in younger dogs.^26^ Our results might be due to higher antibody titers associated with multiple rabies boosters in older dogs and older dogs being more likely to have received at least one vaccine. This finding highlights the effects of population turnover on decreased herd immunity and the importance of boosters in young adult animals to achieve real long-lasting immunity.^6^^,^^7^^,^^49^^,^^50^ Dog population parameters could be useful to assess if young dogs with a single-dose vaccine (Supplemental Table 3) could be boostered with biannual campaigns^51^^,^^52^ or if biannual campaigns are needed to reach new puppies born between campaigns. In some areas, more than 80% of the owners do not know the age at which dogs should receive their first vaccine.^53^ In the neighboring city of Arequipa, where rabies is endemic, 50% of the rabies-positive dogs were less than 1 year old (unpublished data). This information suggests that rabies vaccination in dogs should start as early as possible and strategies to reduce the vaccination gap in puppies should be explored. Even though interference of maternal rabies antibodies may be population specific (e.g., Tanzanian mother dogs may be less likely to be vaccinated compared with dogs in other regions),^7^ evidence strongly suggests a change in the rabies vaccine schedules to include puppies under 3 months old in the mass vaccination campaigns.

Some studies have found that females present higher seroconversion than males,^6^^,^^26^^,^^45^ that small and medium size breeds have higher antibody titers compared with large breeds,^49^ and mutts have higher antibody titers than purebreds.^46^^,^^49^^,^^51^ By contrast, Yale et al. found that purebreds are more likely to seroconvert.^54^ We found similar trends to other previous studies as well (Figure 1D), but these trends were not statistically significant. One potential explanation for the higher rabies antibody titers found in females and small/medium dogs is that they are easier to handle and therefore are vaccinated more frequently than larger or male dogs. It is likely that we did not find statistical significance for these associations because our study was underpowered to analyze those associations. Another limitation of our study is that it was conducted in a central urban area, where a higher proportion of owned dogs are restricted (e.g., they are not free-roaming dogs), compared with peri-urban areas. Also, access to mass vaccination campaigns seems to be higher in urban areas compared with peri-urban areas,^55^ which could influence population immunity. An additional limitation of the study was likely selection bias of the study subjects, which were selected from vaccination sites with the highest influx of dogs rather than randomly chosen. Finally, we did not record the number of previous doses of rabies vaccines, which would have allowed us to analyze the effect of these revaccinations on the immune status of the dogs. The seropositive dog in our study population whose owners reported that it was never vaccinated suggests the possibility of recall bias or the presence of some study subjects with unreliable vaccination history.

Lima, a city with more than 11 million people in its metropolitan area, is considered free of dog rabies virus transmission and has not had any detected cases since 1999. However, the designation free of rabies status might be threatened by current evidence. Our results do not show an improvement in the titer of neutralizing antibodies compared with previous studies that reported insufficient population immunity.^44^ Also, the lack of detected cases could be due to suboptimal number of brain samples submitted for surveillance from some areas of Lima that do not reach the required canine vaccination coverage.^2^^,^^33^^,^^56^ Therefore, vaccination strategies must be reevaluated to replicate the high coverage levels achieved in the 1990 s, adapting to the current context.^28^^,^^57^^,^^58^ Our study provides an important and timely glimpse to the immunity status of the dog population in urban areas of Lima, Peru. Further studies of peri-urban dogs, a larger and more free-roaming population, are needed to obtain a more complete picture of the risk of rabies reestablishment in the capital of Peru.

## Supplemental Material


Supplemental materials
