# Supplementary material for: Dog Population Rabies Immunity before a Mass Vaccination Campaign in Lima, Peru: Vulnerabilities for Virus Reestablishment
Source: Am J Trop Med Hyg. 2023 Jul 10;109(2):420–8. doi: 10.4269/ajtmh.22-0530 (PMC10397462; doi:10.4269/ajtmh.22-0530)
Supplement: Supplementary file 1 [file tpmd220530.SD1.pdf]

Table S1. Geometric mean titers in dogs from Surquillo, Lima - Peru, 2019

Geometric mean titer	n	GMT (95 % CI) IU/mL
Total dogs sampled	141	0.69 (0.17 - 1.21)
Dogs with vaccination history	111	1.39 (0.80 - 1.99)
Dogs without vaccination history	30	0.05 (0 - 0.10)

Table S2. Distribution of antibody titers measured with the FAVN test in dogs from Surquillo, Lima – Peru, 2019.

Category	Antibody titer (IU/mL)	Number of samples (%)
Not previously vaccinated (n=30)	0.03	14 (9.93)
	0.06	12 (8.51)
	0.10	3 (2.13)
	0.87	1 (0.71)
Previously vaccinated (n=111)	0.03 - 0.49	29 (20.57)
	0.5 - 1	15 (10.64)
	1 -2 (1.51)	4 (2.84)
	2 - 4 (2.62)	20 (14.18)
	4 -6 (4.56)	10 (7.09)
	6 - 8 (7.92)	33 (0.10)
	Total	141 (100.00)

Table S3. Characterization of rabies titers measured with FAVN test in dogs with a single vaccine dose, from Surquillo, Lima, Peru, 2019

Rating criteria	Adequate levels (≥0.5 IU/mL)	Non - Adequate levels (<0.5 IU/mL)
Observations	n=17	n=10
Geometric mean titer (IU/mL)	3	0.10
Standard deviation (IU/mL)	3.06	0.09
Median (IU/mL)	4.56	0.135
Minimum value (IU/mL)	0.50	0.03
Maximum value (IU/mL)	7.92	0.29
